# Production of a chimeric porcine reproductive and respiratory syndrome virus (PRRSV)-2 vaccine using a lab-scale packed-bed bioreactor CelCradle

**DOI:** 10.1186/s12917-023-03659-4

**Published:** 2023-08-02

**Authors:** Hwi-Yeon Choi, Jong-Chul Choi, Yeong-Lim Kang, So-Hyeun Ahn, Sang-Won Lee, Seung-Yong Park, Chang-Seon Song, In-Soo Choi, Joong-Bok Lee

**Affiliations:** 1 Laboratory of Infectious Diseases, College of Veterinary Medicine, Konkuk University, 120 Neungdong-ro, Gwangjin-gu, Seoul, 05029 Republic of Korea; 2 KU Research Center for Zoonosis, 120 Neungdong-ro, Gwangjin-gu, Seoul, 05029 Republic of Korea

**Keywords:** Porcine Reproductive and Respiratory Syndrome Virus (PRRSV), Vaccine production, CelCradle, Packed Bed, Bioreactor, Vaccine efficacy

## Abstract

**Background:**

We developed a MARC-145 cell culture and porcine reproductive and respiratory syndrome (PRRS) vaccine production using a novel CelCradle bioreactor. CelCradle is a packed-bed bioreactor capable of both batch and perfusion culture, and the operating parameters are easy to optimize.

**Results:**

In this study, CelCradle reached a maximum cell density of 8.94 × 10^5^ cells/mL at 5 days post-seeding when seeded at 8.60 × 10^4^ cells/mL (doubling time = 35.52 h). Inoculation of PRRS vaccine candidate, K418DM1.1, was performed at a multiplicity of infection (MOI) of 0.01 at 5 days post-seeding, which resulted in a high viral titer of 2.04 × 10^8^ TCID_50_/mL and total viral load of 1.02 × 10^11^ TCID_50_/500 mL at 2 days post-infection (dpi). The multilayer cultivation system, BioFactory culture, yielded a higher doubling time (37.14 h) and lower viral titer (i.e., 8.15 × 10^7^ TCID_50_/mL) compared to the CelCradle culture. Thus, the culture medium productivity of the CelCradle culture was 2-fold higher than that of the BioFactory culture. In the animal experiment, the CelCradle-produced vaccine induced high levels of neutralizing antibodies and effectively protected pigs against homologous challenge, as shown by the significantly lower levels of viremia at 1- and 7-days post-challenge (dpc) compared to the non-vaccinated pigs.

**Conclusions:**

Overall, this study demonstrates that the CelCradle system is an economical platform for PRRS vaccine production.

## Background

The porcine reproductive and respiratory syndrome (PRRS) causes significant productivity losses in the swine industry worldwide [1, 2]. The causative agent of the disease is porcine reproductive and respiratory syndrome virus (PRRSV) which includes Betaarterivirus suid 1 (former PRRSV-1) and Betaarterivirus suid 2 (former PRRSV-2) [3]. Vaccination represents a common and practical tool for PRRS control in affected farms [4, 5]. We recently developed a PRRSV-2 vaccine candidate, K418DM1.1 [6], which is a chimeric virus with hypo-glycosylated glycoprotein 5 (GP5). It is based on the backbone of a FL12 infectious clone [7], containing structural protein genes of the LMY strain [8]. K418DM1.1 was protective against homologous and heterologous viruses under experimental and field conditions [6]. In addition, K418DM1.1 was safe in that no virulent reversion was detected [6]. We demonstrated that K418DM1.1 is a promising vaccine candidate based on its safety and protective efficacy [6].

Once a vaccine candidate is developed, it is important to find a suitable bioreactor system and mass-produce the product in a cost-effective manner. Traditional cell-culture systems, such as roller bottles and multilayer cultivation systems, remain an economic platform for PRRS vaccine production [9, 10]. Bioreactors, however, have advantages over conventional methods in terms of expanded volume, reduced cost, and increased process control [11].

Presently, large-scale microcarrier culture is routinely used for vaccine production in bioreactors [11, 12]. Microcarrier technology has also been applied to the PRRSV production, resulting in successful viral yields [13–15]. Disposable packed-bed bioreactor systems are a good alternative to microcarrier suspension cultures in terms of reducing shear stress [11]. Packed-bed systems are also known to provide large surface areas, enabling high cell concentrations and viral yields [16–18].

We aimed to investigate the feasibility of PRRS vaccine production using CelCradle, a lab-scale packed-bed bioreactor previously known as BelloCell. Previous studies have shown that both cell and product yields of BelloCell were higher than those obtained in roller bottles or microcarrier cultures [19, 20]. The CelCradle was further refined from BelloCell to enable perfusion culture. We compared cell concentrations and viral yields of the platform with those of the conventional multilayer system. Further, we performed an animal experiment using pigs to confirm that the CelCradle-produced vaccine is effective against homologous challenge.

## Results

### Preliminary tests

**Fig. 1:**
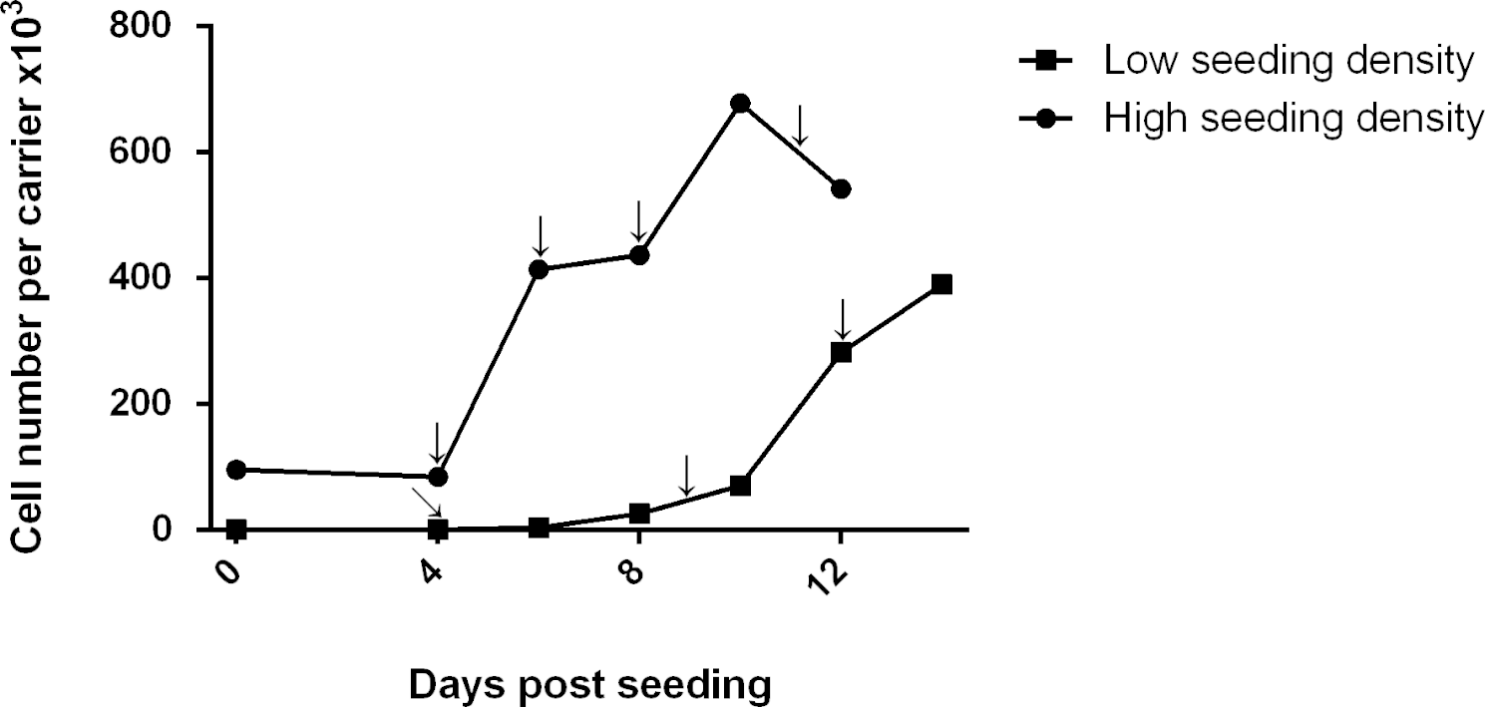
Results of preliminary tests: cell growth of different seeding densities. Arrows indicate the timing of medium exchange

In preliminary tests, the medium was exchanged based on the color at 4-, 9-, and 12-days post-seeding for low seeding densities, and at 4-, 6-, 8-, and 11-days post-seeding for high seeding densities. The maximum cell number was 3.90 ± 0.88 × 10^5^ cells per carrier at 14 days post-seeding at low seeding densities and 6.78 ± 0.75 × 10^5^ cells per carrier at 10 days post-seeding at high seeding densities (Fig. 1).

### Lot A

**Fig. 2:**
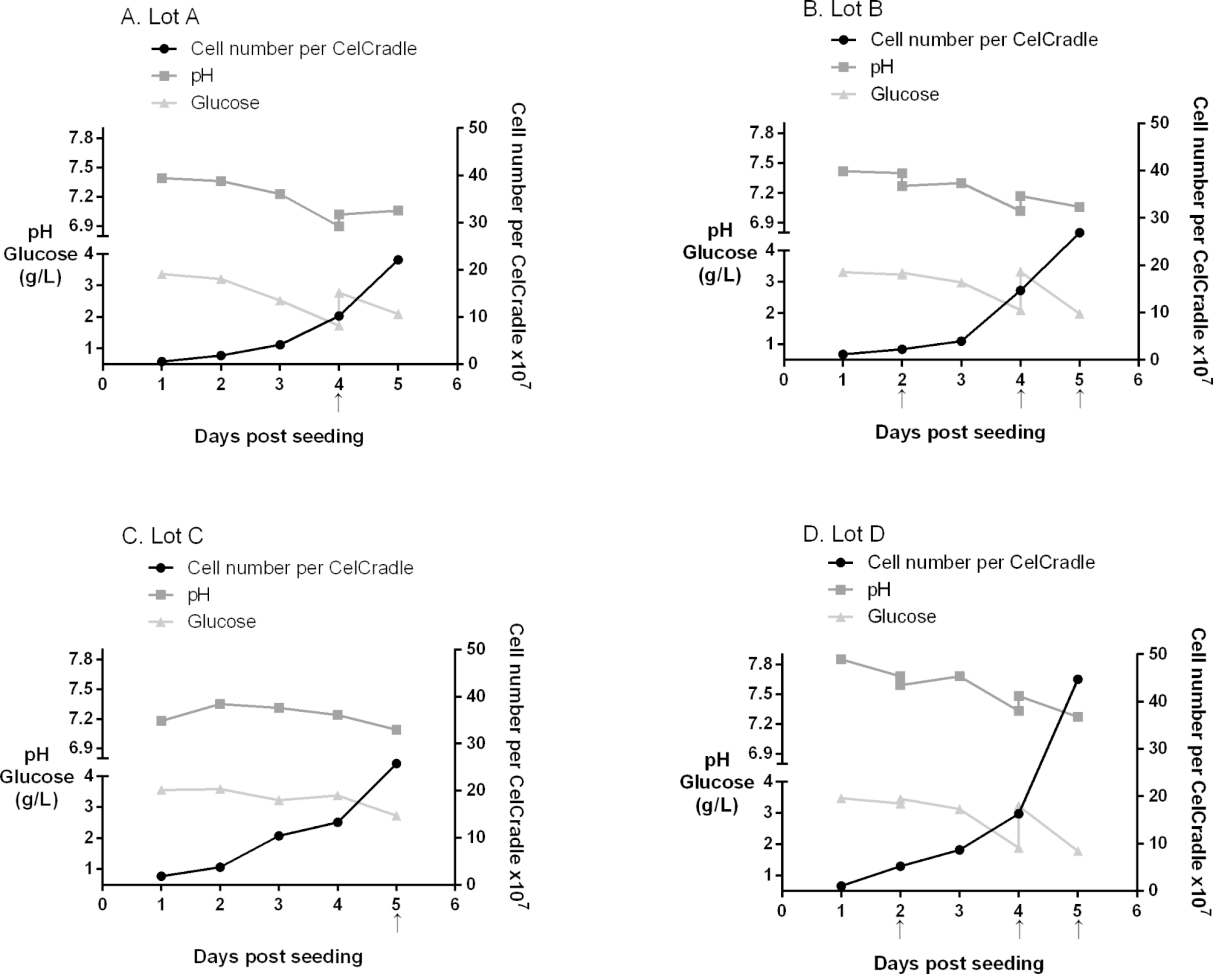
Cell growth, pH, and glucose concentrations of CelCradle. Arrows indicate the timing of medium exchange. (**A**) Lot (A) (**B**) Lot (B) (**C**) Lot (C) (**D**) Lot D

**Fig. 3:**
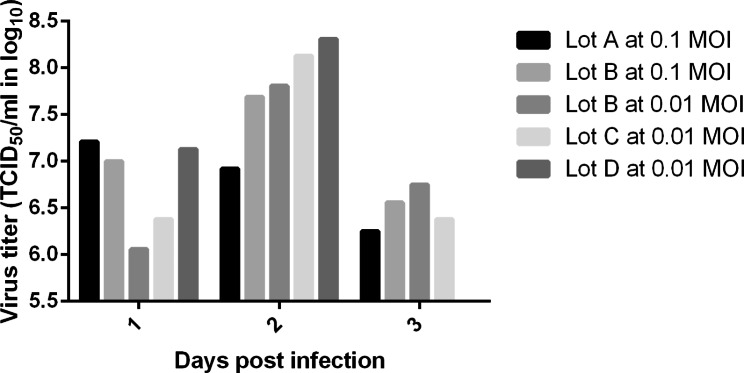
PRRSV production for samples from CelCradle culture collected at indicated days post infection (dpi). The viral titer was expressed as TCID_50_/ml in log_10_

In lot A, the maximum cell number/bottle was observed at 5 days post-seeding (i.e., 2.21 ± 0.95 × 10^8^) (Fig. 2A). The maximum cell number/bottle was 5-fold higher than the inoculum, with a cell doubling time of 51 h (growth rate 0.0136 h^-1^). Complete medium exchange was performed at 4 days post-seeding. The level of glucose was maintained above 1 g/L, but the pH level at 4 days post-seeding was 6.9, which was out of the target range (7.0–7.9). Virus inoculation was performed at a multiplicity of infection (MOI) of 0.1 at 5 days post-seeding (Table 1). Lot A was designed to perform a single harvest of the virus at 3 days post-infection (dpi), without medium exchange after virus inoculation. The maximum viral titer of lot A was 1.62 ± 1.19 × 10^7^ 50% tissue culture infectious dose (TCID_50_)/mL at 1 dpi, and the viral titer decreased to < 10^7^ TCID_50_/mL from 2 dpi (Fig. 3).

**Table 1:** Experimental conditions for cell and viral culture in CelCradle

Run	Medium (cDMEM)	Cell culture	Medium exchange timing during cell culture	Viral culture	Harvest timing during viral culture	Multiplicity of infection (MOI)
Lot A	HyClone DMEM	Semi-batch culture	4 days post-seeding	Fed-batch culture	3 days post-infection (dpi)	0.1
Lot B	HyClone DMEM	Semi-batch culture	2, 4, and 5 days post-seeding	Semi-batch culture	1, 2, and 3 dpi	0.1 and 0.01
Lot C	HyClone DMEM	Perfusion culture	5 days post-seeding	Semi-batch culture	1, 2, and 3 dpi	0.01
Lot D	Welgene DMEM	Semi-batch culture	2, 4, and 5 days post-seeding	Semi-batch culture	1 and 2 dpi	0.01

### Lot B

In lot B, the cell number/bottle peaked at 5 days post-seeding, which was 2.69 ± 1.28 × 10^8^ (Fig. 2B). The maximum cell number/bottle was 6-fold higher than the inoculum, and the cell doubling time was 45 h (growth rate 0.0153 h^-1^). Half of the medium was exchanged at 2 days post-seeding, and the full medium was exchanged at 4- and 5-days post-seeding. Both glucose and pH levels remained within the target ranges. We inoculated the virus at an MOI of 0.1 and 0.01 at 5 days post-seeding (Table 1). Lot B was designed to perform daily harvests of the virus to obtain 500 mL of viral supernatant every day. The viral titer peaked at 2 dpi; the 0.1 MOI infection resulted in 4.90 ± 0.87 × 10^7^ TCID_50_/mL, and the 0.01 MOI infection resulted in 6.46 ± 0.25 × 10^7^ TCID_50_/mL (Fig. 3).

### Lot C

Lot C was performed using a perfusion culture, with a perfusion bottle attached to the batch bottle at 1 day post-seeding. The maximum cell number/bottle was 2.58 ± 1.06 × 10^8^ at 5 days post-seeding (Fig. 2C), which was 6-fold higher than the inoculum, with the cell doubling time of 46 h (growth rate 0.0149 h^-1^). The medium was completely exchanged at 5 days post-seeding. The levels of glucose and pH were maintained within the target ranges. At 5 days post-seeding, we detached the perfusion bottle and inoculated the virus at an MOI of 0.01 (Table 1). Lot C was harvested daily for 500 mL of viral supernatant, which resulted in a maximum viral titer of 1.35 ± 0.47 × 10^8^ TCID_50_/mL at 2 dpi (Fig. 3).

### Lot D

Lot D displayed maximum cell number/bottle at 5 days post-seeding (i.e., 4.47 ± 0.63 × 10^8^) (Fig. 2D; Table 2). The maximum cell number/bottle was 10-fold larger than the inoculum and the cell doubling time of 36 h (growth rate 0.0195 h^-1^) was the shortest of four lots. We exchanged half of the medium at 2 days post-seeding and the full medium at 4- and 5-days post-seeding. The levels of glucose and pH were maintained in appropriate ranges. We performed virus inoculation at 5 days post-seeding at an MOI of 0.01, and obtained 500 mL of the viral supernatant every day by daily harvesting (Table 1). The maximum viral titer was 2.04 ± 0.58 × 10^8^ TCID_50_/mL at 2 dpi (Fig. 3; Table 2), showing the highest viral titer of all lots performed. The total viral yield of lot D was 1.02 × 10^11^ TCID_50_/500 mL.

**Table 2:** Comparison of cell and viral growth using CelCradle and BioFactory

	CelCradle (Lot D)	BioFactory (1-layer)
Cell seeding density (10^4^ cells/ml)	8.60	14.80
Cell doubling time (h)	35.52	37.14
Maximum cell number (10^8^ cells/bottle or 10^8^ cells/layer)	4.47 ± 0.63	1.33 ± 0.52
Maximum cell concentration (10^5^ cells/ml)	8.94 ± 1.26	8.87 ± 3.47
Cell number at time of infection (10^5^ cells/ml)	8.94 ± 1.26	8.87 ± 3.47
Multiplicity of infection (MOI)	0.01	0.01
Viral titer (10^7^ TCID_50_/ml)	20.40 ± 5.80	8.15 ± 0.79
Total virus production (10^10^ TCID_50_/bottle or 10^10^ TCID_50_/layer)	10.20 ± 2.90	1.02 ± 0.10
Cell-specific infectious virus yield (TCID_50_/cell)^a^	228	77

^a^ Cell-specific infectious virus yield was calculated from total virus production and maximum cell number

### BioFactory culture

The BioFactory showed the maximum cell number at 4 days post-seeding (i.e., 1.33 ± 0.52 × 10^8^), which was 6-fold larger than the inoculum, with a cell doubling time of 37 h (growth rate 0.0187 h^-1^) (Table 2). Virus inoculation was performed at 4 days post-seeding at an MOI of 0.1 and 0.01. At 2 dpi, the viral titer of BioFactory reached 7.59 ± 0.26 × 10^7^ TCID_50_/mL for a 0.1 MOI, and 8.15 ± 0.79 × 10^7^ TCID_50_/mL for a 0.01 MOI. BioFactory infected at a 0.01 MOI produced 1.02 × 10^10^ TCID_50_/125 mL of virus in total (Table 2).

The total virus production of one CelCradle bottle of lot D was equal to that of 10 BioFactory layers, and the cell-specific infectious virus yield and culture medium productivity of the CelCradle culture was 3-fold and 2-fold higher than that of the BioFactory culture, respectively (Tables 2 and 3).

**Table 3:** Productivity comparison between CelCradle and BioFactory in producing the PRRS vaccine

Production system	Working units per total virus production of 1.02 × 10^11^ TCID_50_	Total virus production (10^11^ TCID_50_)	Total spent medium (L)				Total process time (d)				Culture medium productivity (10^9^ TCID_50_/L/d)^a^
			Cell inoculum preparation	Cell culture phase	Viral culture phase	Total	Cell inoculum preparation	Cell culture phase	Viral culture phase	Total	
CelCradle (Lot D)	1	1.02	0.00	1.25	1.00	2.25	0	5	2	7	6.48
BioFactory (1-layer)	10	1.02	0.35	1.50	1.25	3.10	4	4	2	10	3.29

^a^ Culture medium productivity was calculated based on Eq. (1) shown in Methods Section

### Protective efficacy of CelCradle-produced vaccine under experimental conditions

After the challenge at 42 days post-immunization, both groups 1 and 2 displayed no significant differences in clinical signs, including the average daily weight gain and rectal temperature.

**Fig. 4:**
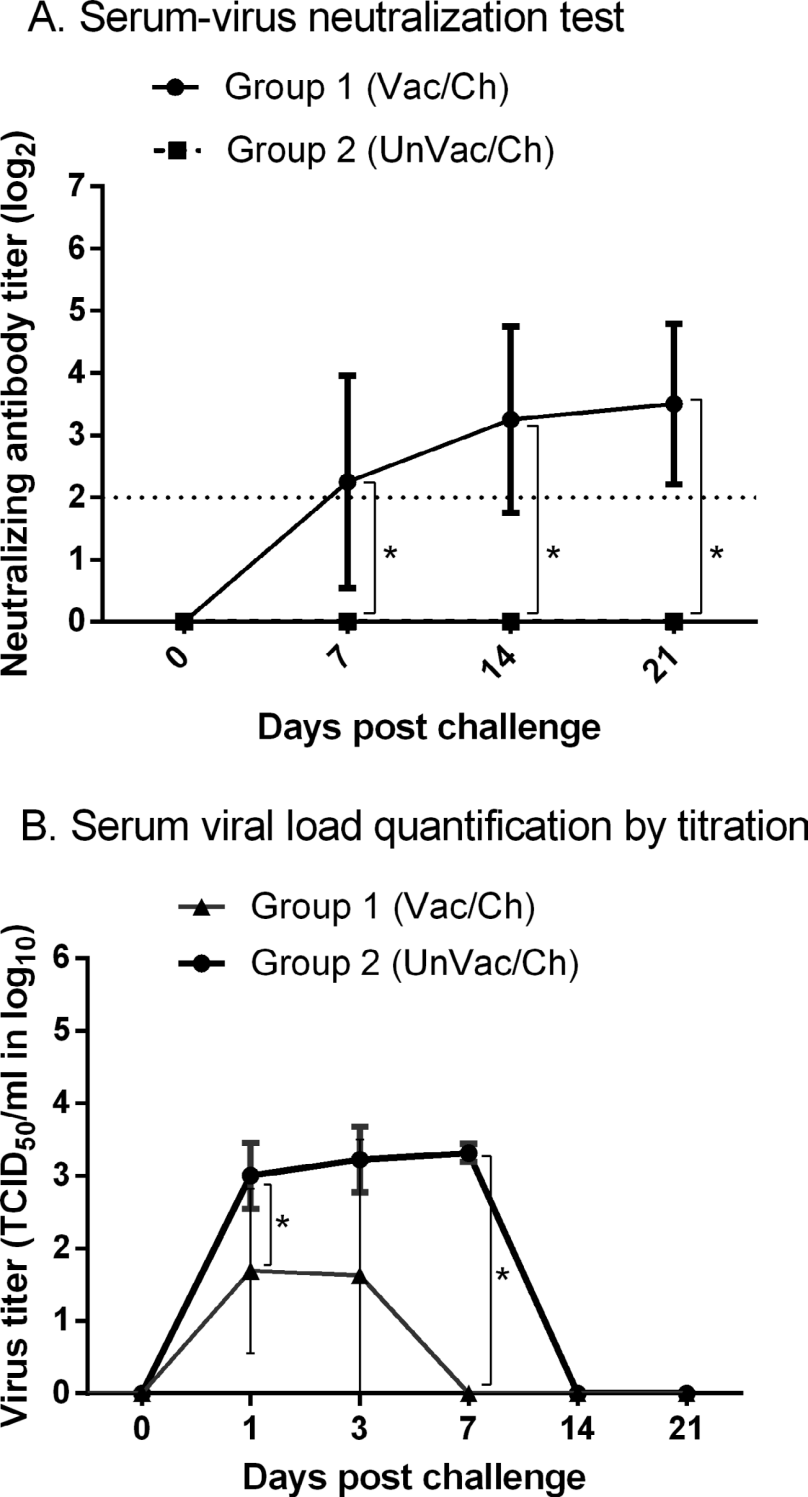
Results of animal experiment: protective efficacy of CelCradle-produced vaccine under experimental conditions. A total of 8 3-week-old pigs were randomly divided into two groups of four each. Group 1 was immunized intramuscularly (IM) with CelCradle-produced K418DM1.1 (10^4.5^ TCID_50_/1 mL/dose), whereas group 2 remained unvaccinated. At 42 days post immunization, both groups were challenged IM with homologous strain, LMY (10^5.0^ TCID_50_/1 mL/dose). (**A**) Neutralizing antibody titer measured against the challenge strain, LMY. Asterisks indicate significant (p ≤ 0.05) differences between the groups. Horizontal dotted line represents the cutoff value of the test. (**B**) Level of viremia measured by titration. Asterisks indicate significant (p ≤ 0.05) differences between the groups

According to the serum-virus neutralization (SVN) test results (Fig. 4A), the vaccinated challenged group (group 1) exhibited significantly (p ≤ 0.05) higher SVN antibody titers than the unvaccinated challenged group (group 2) at 7, 14, and 21 days post-challenge (dpc). The vaccinated challenged group displayed SVN antibody titers of 2.3 ± 1.7 (log_2_), 3.3 ± 1.5 (log_2_), and 3.5 ± 1.3 (log_2_) at 7, 14, and 21 dpc. The unvaccinated challenged group had no detectable SVN antibodies, i.e., < 2 (log_2_), at all time points tested.

Based on the virus titration results of 7 dpc (Fig. 4B), viremia was not detected in all pigs in the vaccinated challenged group, while all pigs in the unvaccinated challenged group displayed high levels of viremia. The viremia level was significantly (p ≤ 0.05) higher in the unvaccinated challenged group than the vaccinated challenged group at 1 and 7 dpc (3.0 ± 0.5 vs. 1.7 ± 1.1; 3.3 ± 0.1 vs. 0.0 ± 0.0). The viremia area under the curve (AUC) from 0 to 21 dpc was also significantly (p ≤ 0.05) higher in the unvaccinated challenged group than in the vaccinated challenged group (6.477 ± 0.308 vs. 1.482 ± 0.996). There was a high correlation between the SVN titers at 21 dpc and the viremia AUC from 0 to 21 dpc (rho = − 0.913, p ≤ 0.05).

Both groups had no detectable viral load in the lungs and tracheobronchial lymph nodes at 21 dpc by titration.

## Discussion

### Preliminary tests

Based on the preliminary test results, we confirmed that MARC-145 cells can attach to the carrier and grow in the carrier without a coating medium such as fibronectin, collagen, or gelatin. Second, we found that one carrier, with its flake-like structure, can house 6.8 × 10^5^ MARC-145 cells. Lastly, we confirmed that it is technically possible to acquire an adequate cell number of 2.4 − 3.0 × 10^5^ cells per carrier from a low seeding density strategy. The low seeding density strategy reached an adequate cell number at 12 days post-seeding. The high seeding density strategy was efficient and an adequate cell number was acquired at 6 days post-seeding. However, a large amount of inoculum is needed for the high seeding density strategy, which is a major hindrance in preparing the cell inoculum or master cell bank for the bioreactor system. Accordingly, we determined the seeding density for CelCradle bioreactor as 5.0 × 10^4^ cells per carrier or 4.3 × 10^7^ cells per CelCradle bottle (850 carriers), which can be reasonably obtained from a routine subculture.

### PRRS vaccine production using CelCradle

Lots A, B, C, and D were conducted to optimize the production of the PRRSV vaccine candidate, K418DM1.1 by a CelCradle bioreactor. During the cell culture phase in lot A, medium exchange was performed at 4 days post-seeding to simulate the routine subculture interval of 3–4 days. However, the cells showed an abrupt growth in the CelCradle bottle at 4 days post-seeding, which led to an unstable pH at 4 days post-seeding. Therefore, in lot B, we added a half medium exchange at 2 days post-seeding before the whole medium exchange at 4 days post-seeding. This additional procedure was designed to simultaneously remove the waste of unattached cell debris and provide supplementary nutrients, thereby supporting rapid cell growth. At 4 days post-seeding, lot B showed stable pH and higher cell growth of 3.7-fold than the cell growth of lot A of 2.5-fold. The higher cell growth at 4 days post-seeding also resulted in a higher maximum cell number at 5 days post-seeding.

To compare the semi-batch and perfusion cultures, the cell culture phase of lot C was performed using the perfusion culture. The maximum cell number/bottle of lot C was not higher than that of the semi-batch culture, i.e., lot B. On the other hand, lot C exhibited better maintenance of pH and glucose concentration than the semi-batch culture. This stability in pH and glucose concentration probably owes to the 2.7 L of working volume in the perfusion culture, which is larger than 0.5 L of that in the batch culture. Hence, the perfusion culture does have an advantage over the semi-batch culture because the stability of pH and glucose concentration in the perfusion culture reduces the labor of periodic medium change.

In the cell culture phase of lot D, we intended to minimize the expenses by choosing the semi-batch culture because the perfusion culture used a 2.2-fold medium than the semi-batch culture. In addition, lot D was performed using different culture media from lots A, B, and C. The composition of the medium was identical, but the medium of lot D had higher pH (7.9 vs. 7.4) and lower price (i.e., US $11.6 vs. US $22.3). Lot D displayed the largest cell number/bottle and shortest cell doubling time among the four lots. The cell doubling time of lot D was also shorter than that of the BioFactory culture. It is generally known that pH levels of 7.2–7.4 are the most appropriate for cell growth. However, we assume that the MARC-145 cells used in this study had been optimized to the medium with a high pH during subculture processes. This implies that when researchers are optimizing the settings for bioreactors, maintaining an identical medium as the subculture can be helpful.

In lot A, at the viral culture phase, we did not exchange the medium from 0 dpi until the virus was harvested at 3 dpi to concentrate the total virus production in the 500 mL medium. However, the maximum viral titer of lot A was lower than that of the BioFactory culture. Hence, in lot B, we exchanged the medium every day from 0 dpi (i.e., 5 days post-seeding) so that the cell and viral growth was supported by the replenished medium. Lot B infected at 0.1 MOI displayed a 3.0-fold higher maximum viral titer than lot A, and lot B infected at 0.01 MOI exhibited a 4.0-fold higher maximum viral titer than lot A. Based on lot B results, we confirmed that the viral yield was best when the CelCradle bottle infected at 0.01 MOI was replenished daily with a fresh medium. Accordingly, lots C and D were infected at an MOI of 0.01, daily harvested for 500 mL of viral supernatant, and provided with 500 mL of fresh medium.

For further optimization, the CelCradle stage moving rate of lot C was revised to increase the contact between virus and cell. The virus adsorption phase was performed for 1 h in lots A and B but was increased to 6 h in lot C. In addition, at the phase of virus production, the upper holding time, which was 0 min in lots A and B, was increased to 1 min in lot C. The maximum viral titer of lot C was 2.1-fold higher than that of lot B, and 1.7-fold higher than that of the BioFactory culture.

In lot D, we performed virus inoculation under the same conditions as lot C, but the higher cell number/bottle of lot D led to a higher maximum viral yield. The cell number/bottle of lot D was 1.7 − 2.0-fold higher than that of lots A, B, and C, and the maximum viral titer of lot D was 1.5 − 12.6-fold higher than that of lots A, B, and C. The maximum viral titer of lot D was 2.5-fold higher than that of the BioFactory culture, which led to the CelCradle culture exhibiting higher viral productivity, cell-specific infectious virus yield, and culture medium productivity compared to the BioFactory culture.

YANG et al. reported remarkable cell growth of 50 − 70-fold in 96 h, which equals the cell doubling time of 15.7 to 17.0 h [14]. Apart from the data of YANG et al., the cell doubling time of lot D (36 h) was generally comparable with the results from other research, which ranged from 28.9 to 62.0 h [15, 21]. Although we chose to inoculate the virus at a cell concentration of 4.4 − 8.9 × 10^5^ cells/mL, previous studies have chosen higher cell concentrations (2.0 − 7.0 × 10^6^ cells/mL) for virus inoculation [13–15]. Higher cell concentrations could have yielded higher virus titers, but the virus production was comparable. Berry et al. achieved 0.3 − 5.0 × 10^7^ TCID_50_/mL of virus and YANG et al. reported 0.1 − 2.0 × 10^7^ TCID_50_/mL of viral titer [9, 14]. Other studies have shown various results from 2.5 × 10^7^ to 1.6 × 10^8^ TCID_50_/mL of virus [15, 22]. These findings indicate that CelCradle culture can achieve higher total virus production with lower cell concentrations compared to other platforms. The vertical flow of the culture medium enables highly efficient oxygen transfer and nutrient supply with low shear stress, which makes CelCradle a favorable culture condition for both cell growth and virus production.

We obtained a high viral titer in the present study, but higher viral yields are expected with further experiments testing different cell seeding densities, virus infection timings, MOIs, and CelCradle stage moving rates. For instance, if we use a lower MOI, we can try continuous cultivation strategies that enable multiple harvests in a perfusion culture. Additionally, we plan to improve the attachment efficiency by developing a CelCradle-adapted cell. Although 90% of the cells were attached to the carriers at the seeding day, the cell number per carrier at 1 day post-seeding was lower than expectation. As Vero cells can be adapted to grow in a serum-free medium after several passages [20, 23], the MARC-145 cells are expected to attach to the carriers better after serial passages in the CelCradle.

### Protective efficacy of CelCradle-produced PRRS vaccine

We evaluated the protective effect of the CelCradle-produced K418DM1.1 vaccine against homologous challenge in pigs. Typically, a high viremia is expected in the PRRSV infection, but effective vaccines can significantly reduce viremia after challenge [6, 24, 25]. In the present study, the vaccine produced by CelCradle significantly reduced viremia after the challenge, which demonstrates the efficacy of the vaccine. According to a previous study, the K418DM1.1 vaccine produced by BioFactory culture exhibited a viremia AUC of 0.655 ± 1.245 after homologous challenge [6]. No significant differences (p > 0.05) were found between the viremia AUC of the two vaccine production platforms. It has also been reported that efficacious vaccination can induce a 25% reduction in viremia AUC [25]. The vaccine produced by CelCradle and BioFactory cultures induced 77% and 87% reduction in viremia AUC, respectively [6], which shows that K418DM1.1 is effective regardless of the vaccine production platforms.

We performed SVN tests to investigate the relationship between SVN antibodies and vaccine efficacy. In our study, the vaccinated challenged group displayed high SVN titers, and a high correlation was detected between the SVN titers and the viremia AUC. These results are consistent with a previous study showing that the K418DM1.1 vaccine produced by the BioFactory culture induced high SVN titers which were highly correlated with the viremia AUC [6]. Thus, the results suggest that humoral immunity contributes to the protective performance of the K418DM1.1 vaccine produced by CelCradle, as in the vaccine produced by the BioFactory culture.

## Conclusions

The present study demonstrates that CelCradle can serve as a suitable platform for the MARC-145 cells and PRRSV culture. The CelCradle culture showed improved viral productivity and culture medium productivity than the traditional cell-culture system, the BioFactory culture. In the animal experiment, the efficacy of the K418DM1.1 vaccine produced by CelCradle was comparable to that of the vaccine produced using the BioFactory culture.

We illustrated the detailed process of optimization of CelCradle production to emphasize the simplicity of the platform. Our data can help other researchers interested in using the CelCradle system, providing an optimization example. The culture protocol of the CelCradle can be directly translated to industrial-scale bioreactors with working volume ranging from 10 to 5,000 L [26, 27]. Therefore, CelCradle holds great potential for the commercial production of vaccines and biologicals.

## Methods

### Cell, medium, and virus

The MARC-145 cell line, which is a highly PRRSV-susceptible subpopulation of the African green monkey kidney MA-104 cell line, was used in this study [28]. A PRRSV vaccine candidate, K418DM1.1, was reverse genetically generated by our research team in a previous study [6] and inoculated into MARC-145 cells. For cell and viral cultures, high-glucose (4,500 mg/L) Dulbecco’s modified Eagle’s medium (DMEM; Cat#LM001-05, Welgene; Cat#SH30243.01, HyClone) supplemented with 25 mM HEPES (Cat#15630-080, Gibco), 10% fetal bovine serum (Cat#12483-020, Gibco), and 1% antibiotic/antimycotic solution (Cat#15240-062, Gibco) was used as the complete DMEM medium (cDMEM). cDMEM of lot A, B, and C were made from HyClone DMEM, while cDMEM of lot D and BioFactory (NEST Biotech, China) culture were made from Welgene DMEM. All cultures were maintained at 37 °C in a humidified atmosphere of 5% CO_2_. Experiments were performed three times, and the data were expressed as mean ± standard deviation (SD).

### BioNOC II carriers

BioNOC II carriers (non-woven fabric strips, 5-mm width, 10-mm length; Esco Aster, Singapore) were used as the matrix for growing cells. They are made of 100% polyethylene terephthalate (PET) material and provide a surface area of 2,400 cm^2^/g with a high surface to volume ratio (S/V) of 160 cm^− 1^. Carriers are either prepacked in a CelCradle bottle (Esco Aster, Singapore) by a unit of 850 pieces and pre-sterilized by γ-irradiation or individually packed in a unit of 30 pieces in a pre-sealed bag for small-scale experiments. The individually packed carriers were submerged in phosphate-buffered saline (PBS; Cat#P4417, Sigma) and autoclaved for long-term storage at room temperature.

### Preliminary tests with BioNOC II carriers

Preliminary tests using 6-well plates were conducted to confirm the viable cell number a carrier can house and form the basis of the CelCradle experimental designs. We strategized two seeding densities, the high seeding density and low seeding density. The high seeding density refers to 9.6 × 10^4^ cells per carrier, which corresponds to 8.2 × 10^7^ cells per CelCradle, and the low seeding density refers to 8.8 × 10^2^ cells per carrier, which corresponds to 7.5 × 10^5^ cells per CelCradle.

Briefly, autoclaved carriers were placed in a 15 mL conical tube with the cell inoculum at pre-determined density. The tube was incubated in a 5% CO_2_ humidified incubator at 37 °C for 3 h with gentle inverting every 15 min. After 3 h, an attachment rate of 90% was confirmed and the carriers were transferred to 6-well plates. The 6-well plates were grown in a 5% CO_2_ humidified incubator at 37 °C for 12–14 days, and cell growth was monitored on alternate days starting from 4 days post-seeding. At every monitoring, we harvested cells from three carriers by trypsinization and performed manual counting by trypan blue dye exclusion method. The medium exchange was performed when the color of the medium turned from orange-red to orange.

### Cell culture in CelCradle

**Fig. 5:**
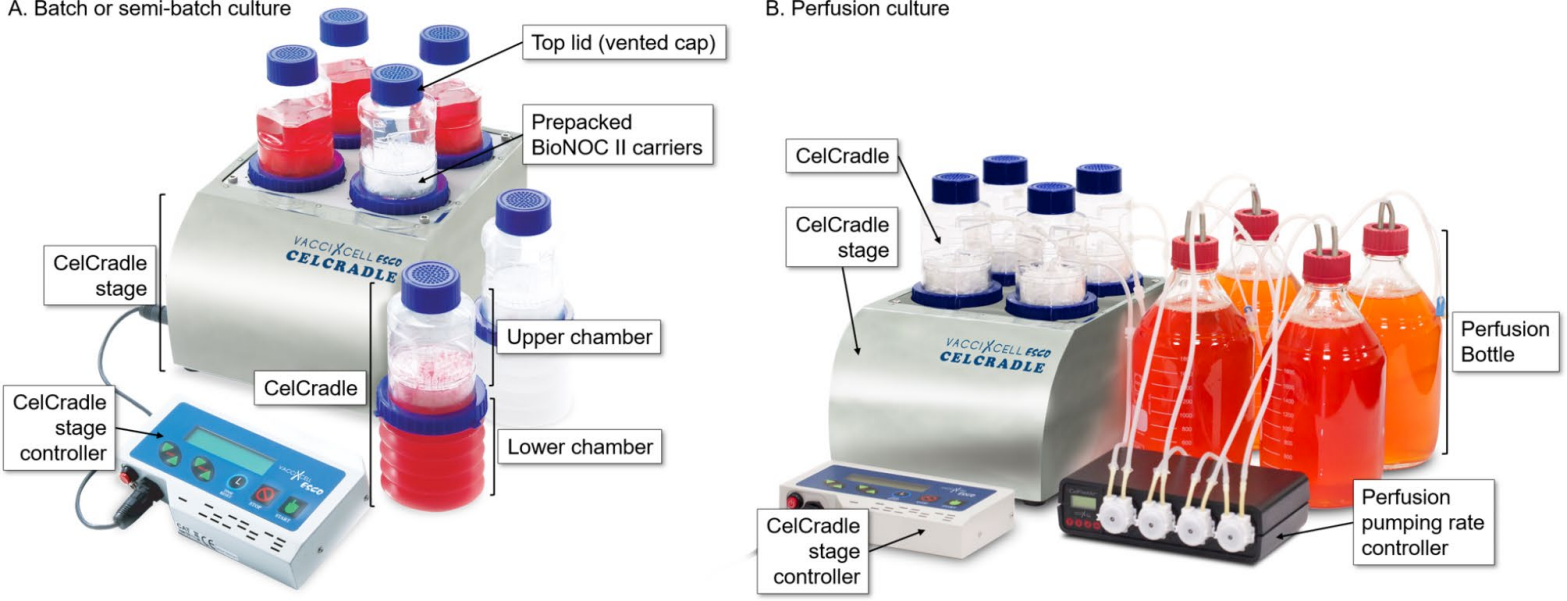
Schematic diagrams of CelCradle system. CelCradle can be operated in batch or perfusion mode. (**A**) Batch or semi-batch culture. (**B**) Perfusion culture

The CelCradle bottle has a working volume of 500 mL and comprises two chambers, the upper and lower chambers (Fig. 5). The upper chamber is prepacked with carriers and serves as a packed bed, while the lower chamber containing the medium is alternately compressed and decompressed by the console system, i.e., CelCradle stage (Esco Aster, Singapore). When the lower chamber is compressed, the medium level rises, and nutrient transfer occurs. The lower chamber is then decompressed to decrease the medium level, allowing oxygen transfer.

The cell culture in the CelCradle bottle was performed as follows. A total of 4.3 × 10^7^ cells were suspended in a 120 mL of medium and transferred to a CelCradle bottle with a non-vented cap. We inverted the bottle so that the carriers were submerged in the cell inoculum. The inverted bottle was incubated in a 5% CO_2_ humidified incubator at 37 °C for 3 h with gentle swirling every 15 min. After 3 h, we confirmed that > 90% of cells were attached to the carriers. Then, we topped up the CelCradle with a 500 mL medium and exchanged the top lid into a vented cap. Finally, the CelCradle bottle was placed on the CelCradle stage with a moving rate of up/down speed of 1.0 mm/s, upper holding time (UH) of 0 min, and down holding time (DH) of 1 min. In the perfusion culture (Fig. 5B), 2.2 L perfusion bottle (Cat#1,112,715, Duran) was attached to the CelCradle bottle at 1 day post-seeding, and the perfusion pumping rate was set at 1,999 mL per day in 24 cycles.

We performed four lots (lots A, B, C, and D) sequentially, reflecting the data of the previous lot to optimize the process. During the cell culture phase, a semi-batch culture was used in lots A, B, and D, while the perfusion culture was used in lot C.

### Virus production in CelCradle

For virus inoculation, the cell number of 2.4 − 3.0 × 10^5^ cells per carrier or 2.0 × 10^8^ cells per CelCradle was used as the parameter of infection. When appropriate cell concentrations were achieved, we inoculated K418DM1.1 virus at an MOI of 0.01 to 0.1.

During the virus absorption phase, the CelCradle stage was maintained at up speed of 2.0 mm/s, UH of 10 min, down speed of 0.25 mm/s, and DH of 1 min. After 1 h of viral absorption, lots A and B were converted to the virus production phase, with a linear moving rate of 1.0 mm/s, UH of 0 min, and DH of 1 min. In lots C and D, the virus absorption phase was maintained for 6 h, and the moving rate of the virus production phase was modified to an up/down speed of 1.0 mm/s with UH/DH of 1 min.

For lot A, we performed a single harvest at 3 dpi. On the other hand, daily harvests were performed in lots B, C, and D for 2–3 days. The harvested virus solution was divided into aliquots and stored at − 70 °C until further analysis.

### Monitoring of cell and viral growth in CelCradle

During cell and viral culture in CelCradle, we measured pH and glucose concentrations every day using a glucometer (Cat#1,400,009, Esco Aster) and pH probe (Thermo Fisher Scientific, USA). The target pH was 7.0–7.9, and the target glucose concentration was above 1 g/L. To maintain appropriate glucose concentrations, we simply added glucose (Cat#A2494001, Gibco) or exchanged the medium. The pH level was maintained by adding sodium bicarbonate (Cat#S5761, Sigma), withdrawing CO_2_, or exchanging the medium.

Cell growth in the CelCradle was monitored every day following the manufacturer’s protocol. Briefly, we sampled three carriers from the bottle and harvested the cells by trypsinization. The cell number was determined using a hemocytometer and trypan blue dye exclusion method.

Viral growth was measured on a daily basis by titration on MARC-145 cells and viral titers were expressed as TCID_50_/mL.

### BioFactory culture

BioFactory (NEST Biotech, China) is a multilayer cultivation system with a growth area of 647 cm^2^ and working volume of 150 mL in a single layer. In the single layer of BioFactory, we seeded a total of 2.2 × 10^7^ cells suspended in a 150 mL of medium. When the cells grew to confluence at 4 days post-seeding, they were harvested by trypsinization for manual counting. The cells were counted using a hemocytometer and trypan blue dye exclusion method.

For virus inoculation, at 4 days post-seeding, the medium was removed, and the virus was inoculated at an MOI of 0.01–0.1. After 1 h, the virus solution was eliminated, and 125 mL of medium was added to the single layer of the BioFactory. The virus was harvested when 80% cytopathic effect (CPE) was observed at 2 dpi. The viral titer of the harvested solution was determined by titration on MARC-145 cells.

### Animal experiment

To assess the protective efficacy of CelCradle-produced vaccine, we conducted a homologous challenge test using pigs. The animal experiment was approved by the Konkuk University Institutional Animal Care and Use Committee (No. KU20056).

A total of 8 three-week-old, crossbred (large white-landrace-duroc triple cross) castrated piglets were obtained from a PRRSV-free herd and randomly divided into two groups of four each. The random number generator (SPSS Inc., Chicago, USA) was utilized to assign the piglets to each group and sample size per each group was determined based on the PRRS vaccine efficacy study designs of previous studies [29–31]. Piglets were acclimatized for 3 days before initiation of the experiments. Group 1 was immunized intramuscularly (IM) on the left side of the neck (needle 23G, 1ʺ long) with 1 mL of 10^4.5^ TCID_50_ of K418DM1.1, which was produced by the CelCradle culture. Group 2 remained unvaccinated and inoculated IM (same condition as above) with 1 mL of PBS. Investigators could not be blinded to the group allocation during the experiments, because vaccinated group was separated from the unvaccinated group to prevent virus shedding. At 42 days post-immunization (0 dpc), both groups were challenged IM (same condition as above) with 1 mL of 10^5.0^ TCID_50_ of the LMY strain, which shares 97% nucleotide sequence identity of the GP5 gene with K418DM1.1. The pigs were observed until 21 dpc and were humanely euthanized by electrocution as described by the guidelines of American Veterinary Medical Association (AVMA) [32]. We monitored rectal temperatures and clinical symptoms at 0, 1, 3, 7, 14, and 21 dpc and recorded body weights at 0, 7, 14, and 21 dpc. To measure the level of viral load, we performed titration on MARC-145 cells using sera collected at 0, 1, 3, 7, 14, and 21 dpc and tissues of the lungs and tracheobronchial lymph nodes collected at 21 dpc. Serum samples collected at 0, 7, 14, and 21 dpc were also evaluated for the level of PRRSV-specific neutralizing antibodies (NAs) by the SVN test. No inclusion or exclusion criteria were used and there were no exclusions of animals, experimental units, or data points.

### Titration

We performed titration on MARC-145 cells to measure the viral titer of the harvested solution and the viral load of sera and tissues as previously described [33].

### SVN test

We performed the SVN tests on MARC-145 cells to measure the amount of PRRSV-specific NAs, as previously described [34]. Briefly, heat-inactivated serum samples were two-fold serially diluted in the medium. An equal volume of the challenge virus, LMY strain, at a concentration of 4 × 10^3.0^ TCID_50_/1 mL, was added to each diluted sample. The mixtures were incubated for 1 h at 37 °C and inoculated onto 96-well plates containing confluent MARC-145 cell monolayers. After 48 h, cells were fixed and stained with anti-PRRSV-2 monoclonal antibody (Cat# NA9041, Median diagnostics), which were then incubated with a secondary antibody labeled with Alexa Fluor 488 (Cat# A11001, Thermo Fisher Scientific). SVN antibody titers were expressed as the last dilution that exhibited a 90% or higher reduction in the number of fluorescent foci.

### Statistical analysis

Researchers were blinded to the group allocation during the statistical analysis. Prior to the statistical analysis, viremia and NAs data were transformed to log_10_ and log_2_ values, respectively. We calculated the AUC of the viremia data using the trapezoidal rule. According to the Shapiro-Wilk test, data were not normally distributed, and differences between the groups were analyzed by the Mann-Whitney U and Kruskal-Wallis tests. We assessed the correlation between viremia AUC and NAs data using Spearman’s rank correlation test. A p-value ≤ 0.05 was considered statistically significant. IBM SPSS Statistics for Windows, Version 25.0. (IBM Corp., Armonk, NY) was used for the statistical analyses, and GraphPad Prism for Windows, Version 6.01 (GraphPad Software, CA, USA) was used for designing the graphs.

### Calculation

The culture medium productivity P_v_ [TCID_50_/L/d] was calculated from the total virus production (v_tot_) [TCID_50_], total spent medium (m_tot_) [L], and total process time (t_tot_) [d], considering cell inoculum preparation, cell culture phase, and viral culture phase (Eq. (1)) as previously described [35] with some modifications.

1 $${P_v} = {v_{tot}}/({m_{tot}} \times {t_{tot}})$$

## Abbreviations

AUC: area under the curve
DH: down holding time
dpc: days post-challenge
dpi: days post-infection
DMEM: Dulbecco’s modified Eagle’s medium
GP5: glycoprotein 5
IM: intramuscularly
MOI: multiplicity of infection
NAs: neutralizing antibodies
PRRS: porcine reproductive and respiratory syndrome
S/V: surface to volume ratio
SVN: serum-virus neutralization
TCID_50_: 50% tissue culture infectious dose
UH: upper holding time
