# Correction: Production of a chimeric porcine reproductive and respiratory syndrome virus (PRRSV)-2 vaccine using a lab-scale packed-bed bioreactor CelCradle

**DOI:** 10.1186/s12917-023-03685-2

**Published:** 2023-08-09

**Authors:** Hwi-Yeon Choi, Jong-Chul Choi, Yeong-Lim Kang, So-Hyeun Ahn, Sang-Won Lee, Seung-Yong Park, Chang-Seon Song, In-Soo Choi, Joong-Bok Lee

**Affiliations:** 1 Laboratory of Infectious Diseases, College of Veterinary Medicine, Konkuk University, 120 Neungdong-ro, Gwangjin-gu, 05029 Seoul, Republic of Korea; 2 KU Research Center for Zoonosis, 120 Neungdong-ro, Gwangjin-gu, 05029 Seoul, Republic of Korea

**Correction: BMC Vet Res 19, 105 (2023)**

10.1186/s12917-023-03659-4

Following publication of the original article [1], the authors identified an error in Table 1 as all of its rows and entries are visible in the web version, but the last two rows are missing in the pdf version.

The original article has been corrected.
